# Supplementary material for: Health Deficits Among People Experiencing Homelessness in an Australian Capital City: An Observational Study
Source: Int J Environ Res Public Health. 2025 Jan 21;22(2):135. doi: 10.3390/ijerph22020135 (PMC11855107; doi:10.3390/ijerph22020135)
Supplement: Supplementary file 1 [file ijerph-22-00135-s001.zip › ijerph-3292674-supplementary.pdf]

International Journal of *Environmental Research and Public Health*

MDPI

Supplementary Table 1. Demographic characteristics, by gender and age group differences

	p value	Male (23-84 yrs)	Female (22-69 yrs)	Total (22-84 yrs)
Age in years (m, SD)		*51.4 (15.2)*	*46.1 (14.3)*	*49.1 (14.9)*
		**n (%)**	**n (%)**	**n (%)**
**Age**	0.384			
Less than 55 years		16 (53.3)	15 (65.2)	31 (58.5)
55 years or over		14 (46.7)	8 (34.8)	22 (41.5)
**Level of education**	0.662			
High school only		18 (60.0)	11 (47.8)	29 (54.7)
Certificate/trade		5 (16.7)	6 (26.1)	11 (20.8)
Diploma/degree		3 (10.0)	4 (17.4)	7 (13.2)
Post graduate		2 (6.7)	2 (8.7)	4 (7.5)
Not reported		2 (6.6)	0 (0.0)	2 (3.8)
**Income**	0.396			
Pension		29 (93.5)	19 (86.4)	48 (90.6)
Wage		2 (6.5)	1 (4.5)	3 (5.7)
Not reported		0 (0.0)	2 (9.1)	2 (3.7)
**Marital status**	**0.034**			
Married/de facto		4 (13.3)	**8 (34.8)**	12 (22.6)
Single, divorced, separated, widowed		**26 (86.7)**	14 (60.9)	40 (75.5)
Not reported		0(0.0)	1 (4.3)	1 (1.9)
**Primary language spoken**	0.213			
English		26 (83.9)	21 (95.5)	47 (88.7)
Language other then English		4 (12.9)	0 (0.0)	4 (7.5)
Not reported		1 (3.2)	1 (4.5)	2 (3.8)

Statistically more likely gender shown in bold; grp: group; m: mean; n: number observed; *p* value: Pearsons Chi Square for normally distributed data; SD: standard deviation of the mean

Supplementary Table 2. Health assessments, by gender and age group differences (significantly more likely highlighted bold)

	Overall	Male		Female		Less than 55 years		55 years and over	
	n/N (%, 95% CI)	n	% (95% CI)	n	% (95% CI)	n	% (95% CI)	n	% (95% CI)
Anthropometry (objective measurement)									
Over recommended waist to hip ratio ¥¥	33/46 (71.7, 56.5 – 84.0)	22	**84.6 (65.1 – 95.6)**	11	55.0 (13.5 – 76.9)	19	65. 5 (45.7 – 82.1)	14	**82.4 (56.6 – 96.2)**
Over recommended waist circumference	31/46 (67.4, 52.0 – 80.5)	18	69.2 (48.2 – 85.7)	13	65.0 (40.8 – 84.6)	20	69.0 (49.2 – 84.7)	11	64.7 (38.3 – 85.8)
Muscle mass under recommended thresholds	27/48 (56.2, 41.2 – 70.5)	14	50.0 (30.6 – 69.3)	13	61.9 (38.4 – 81.9)	19	63.3 (43.8 – 80.1)	8	42.1 (20.2 – 66.5)
BMI – obesity	21/53 (39.6, 26.5 – 54.0)	11	36.7 (19.9 – 56.1)	10	43.5 (23.2 – 65.5)	13	41.9 (24.5 – 60.9)	8	36.4 (17.2 – 59.3)
Audiology (objective measurement)									
Ear health (wax, wax occlusion, pus/discharge)	16/42 (38.1, 23.6 – 54.4)	10	43.5 (23.2 – 65.5)	6	31.6 (12.6 – 56.5)	7	26.9 (11.6 – 47.8)	9	**55.6 (30.0 – 80.2)**
Unknown hearing problem ¥¥	17/49 (34.7, 21.7 – 49.6)	13	**48.1 (28.7 – 68.0)**	4	18.2 (5.2 – 40.3)	8	25.8 (11.8 – 44.6)	9	**50.0 (26.0 – 74.0)**
Diet and nutrition (self-reported)									
Moderate to high nutritional risk	48/53 (90.6, 79.3 – 96.9)	28	93.3 (78.0 – 99.2)	20	87.0 (66.4 – 33.6)	28	90.3 (74.2 – 98.0)	20	90.9 (70.8 – 98.9)
Poor appetite in past week ¥	26/52 (50.0, 35.9 – 64.2)	15	51.7 (32.5 – 70.5)	11	47.8 (26.8 – 69.4)	14	45.2 (27.3 – 64.0)	12	**57.1 (34.0 – 78.2)**
Food insecurity in past week ¥¥	21/50 (42.0, 28.2 – 56.8)	13	**46.4 (27.5 – 66.1)**	8	36.3 (17.2 – 59.3)	11	36.7 (19.9 – 56.1)	10	**50.0 (27.2 – 72.8)**
Water consumption (< than 2L per day) *	19/50 (38.0, 24.7 – 52.8)	10	35.7 (18.6 – 55.9)	9	40.9 (20.7 – 63.6)	10	34.5 (17.9 – 54.3)	9	42.9 (21.8 – 66.0)
Soft drinks (> 0.5L per day) *	16/50 (32.0, 19.5 – 46.7)	11	39.3 (21.5 – 59.4)	5	22.7 (7.8 – 45.4)	8	27.6 (12.7 – 47.2)	8	38.1 (18.1 – 61.6)
Junk food (twice or more per week) *	15/52 (28.8, 17.1 – 43.1)	9	31.0 (15.3 – 50.8)	6	26.1 (10.2 – 48.4)	10	32.3 (16.7 – 51.4)	5	23.8 (8.2 – 47.2)
Other physical health (objective measurement)									
FMS (at risk 3 or more movements) ¥	27/43 (62.8, 46.7 – 77.0)	11	44.0 (24.4 – 65.1)	5	27.8 (9.7 – 53.5)	9	31.0 (15.3 – 50.8)	7	**50.0 (23.0 – 77.0)**
Skin health (≥1 skin condition on inspection)	24/43 (55.8, 39.9 – 70.9)	14	56.0 (34.9 – 75.6)	10	55.6 (30.7 – 78.5)	14	50.0 (30.6 – 69.3)	10	66.7 (38.4 – 88.2)
Obstructive/restrictive/mixed pattern airflow ¥	21/46 (45.7, 30.9 – 61.0)	11	44.0 (24.4 – 65.1)	10	47.6 (25.7 – 70.2)	12	41.4 (23.5 – 61.1)	9	**52.9 (27.8 – 77.0)**
Grip strength (below age and gender norms)	13/45 (28.9, 16.4 – 44.3)	8	33.3 (15.6 – 55.3)	5	23.8 (8.2 – 47.2)	7	25.9 (11.1 – 46.3)	6	33.3 (13.3 – 59.0)
Other physical health (self-reported)									
Overall general health (fair or poor)	16/44 (36.4, 22.4 – 52.2)	8	32.0 (14.9 – 53.5)	8	42.1 (20.3 – 66.5)	7	26.9 (11.6 – 47.8)	9	50.0 (26.0 – 74.0)
At least one health condition	50/53 (94.3, 84.3 – 98.8)	29	96.7 (82.8 – 99.9)	21	91.3 (72.0 – 98.9)	29	93.5 (78.6 – 99.2)	21	95.5 (77.1 – 99.9)
At least one area of PFB	32/51 (62.7, 48.1 – 75.9)	17	58.6 (38.9 – 76.5)	15	68.2 (45.1 – 86.1)	19	61.3 (42.2 – 78.1)	13	65.0 (40.8 – 84.6)
Poor sleep quality (PSQI) ¥	27/48 (56.2, 41.2 – 70.5)	15	53.6 (33.9 – 72.5)	12	60.0 (36.0 – 80.9)	19	**65.5 (45.7 – 82.1)**	8	42.1 (20.2 – 66.5)
Cognition and memory (GP-COG < 8)	26/47 (55.3, 40.1 – 69.8)	15	55.6 (35.3 – 74.5)	11	55.0 (31.5 – 76.9)	17	58.6 (38.9 – 76.5)	9	50.0 (26.0 – 74.0)

Frailty - vulnerable, mildly/moderately frail ¥	23/52 (44.2, 30.5 – 58.7)	14	46.7 (28.3 – 65.7)	9	40.9 (20.7 – 63.6)	11	35.5 (19.2 – 54.6)	12	**57.1 (34.0 – 78.2)**
Fall/near fall in past 6 months ¥	22/51 (43.1, 29.3 – 57.8)	11	39.3 (21.5 – 59.4)	11	47.8 (26.8 – 69.4)	12	38.7 (21.8 – 57.8)	10	**50.0 (27.2 – 72.8)**
Oral health (self-reported)									
Overall dental health (fair or poor)	21/44 (47.7, 32.5 – 63.3)	12	48.0 (27.8 – 68.7)	9	47.4 (24.4 – 71.1)	8	32.0 (14.9 – 53.5)	8	42.1 (20.3 – 66.5)
Dental health worse than overall health **	14/44 (31.8, 18.6 – 47.6)	10	40.0 (21.1 – 61.3)	4	21.1 (6.1 – 45.6)	9	34.6 (17.2 – 55.7)	5	27.8 (9.7 – 53.5)
One or more of OHIP-14 domains affected ¥ǂ	26/44 (59.1, 43.2 – 73.7)	14	56.0 (34.9 – 75.6)	12	63.2 (38.5 – 83.7)	13	50.0 (29.9 – 70.1)	13	**72.2 (46.5 – 90.3)**
Five or more teeth extracted (range 0 – 32) ¥	19/37 (51.4, 34.4 – 68.1	11	52.4 (29.8 – 74.3)	8	50.0 (24.7 – 75.3)	7	35.0 (15.4 – 59.2)	12	**70.6 (44.0 – 89.7)**
Six or more filled teeth (range 0 - 32) ¥	20/44 (45.5, 30.4 – 61.2	13	52.0 (31.3 – 72.2)	7	36.8 (16.3 – 61.6)	7	26.9 (11.6 – 47.8)	13	**72.2 (46.5 – 90.3)**
Oral health (D2) Physical pain ǂ	16/44 (36.4, 22.4 – 52.2)	9	36.0 (18.0 – 57.5)	7	36.8 (16.3 – 61.6)	8	30.8 (14.3 – 51.8)	8	44.4 (21.5 – 69.2)
Oral health (D4) Physical disability ǂ	16/44 (36.4, 22.4 – 52.2)	6	24.0 (9.3 – 45.1)	10	52.6 (28.9 – 75.5)	7	26.9 (11.6 – 47.8)	9	50.0 (26.0 – 74.0)
Oral frailty – less than 20 functioning teeth	12/37 (32.4, 18.0 – 49.8)	7	33.3 (14.6 – 57.0)	5	31.3 (11.0 – 58.7)	3	15.0 (3.2 – 37.9)	9	52.9 (27.8 – 77.0)
Oral health (D7) Handicap ǂ	13/44 (29.5, 16.8 – 45.2)	8	32.0 (14.9 – 53.5)	5	26.3 (9.1 – 51.2)	9	34.6 (17.2 – 55.7)	4	22.2 (6.4 – 47.6)
Oral health (D1) Functional limitation ǂ	9/44 (20.5, 9.8 – 35.3)	6	24.0 (9.3 – 45.1)	3	15.8 (3.4 – 39.6)	5	19.2 (6.5 – 39.3)	4	22.2 (6.4 – 47.6)
Psychological health (self-reported)									
Psychological distress (K10) ¥¥	26/47 (55.3, 40.1 – 69.8)	11	40.7 (22.4 – 61.2)	15	**75.0 (50.9 – 91.3)**	18	**62.1 (42.3 – 79.3)**	8	44.4 (21.5 – 69.2)
Mental health condition ¥	21/53 (39.6, 26.5 – 54.0)	12	40.0 (22.6 – 59.4)	9	39.1 (19.7 – 61.4)	17	**54.8 (36.0 – 72.7)**	4	18.2 (5.2 – 40.3)
Oral health (D3) Psychological discomfort ǂ	16/44 (36.4, 22.4 – 52.2)	8	32.0 (14.9 – 53.5)	8	42.1 (20.2 – 66.5)	11	42.3 (23.3 – 63.1)	5	27.8 (9.7 – 53.5)
Oral health (D5) Psychological disability ǂ	14/44 (31.8, 18.6 – 47.6)	9	36.0 (18.0 – 57.5)	5	26.3 (9.1 – 51.2)	8	30.8 (14.3 – 51.8)	6	33.3 (13.3 – 59.0)
Oral health (D6) Social disability ǂ	8/44 (18.2, 8.2 – 32.7)	4	16.0 (4.5 – 36.1)	4	21.1 (6.0 – 45.6)	6	23.1 (9.0 – 43.6)	2	11.1 (13.7 – 34.7)
Health service use (self-reported)									
ED presentation – past year ***¥	17/53 (32.1, 19.9 – 46.3)	9	30.0 (14.7 – 49.4)	8	34.8 (16.4 – 57.3)	8	25.8 (11.8 – 44.6)	9	**40.9 (20.7 – 63.6)**
Hospital admission – past year ***	16/52 (30.8, 18.7 – 5.1)	9	30.0 (14.7 – 49.4)	7	31.8 (13.9 – 54.9)	8	26.7 (12.3 – 45.9)	8	36.4 (17.2 – 59.3)
Protective health behaviours (self-reported)									
Not undertaken vaccination – past 5 years (flu, meningococcal, pneumonia, shingles) ¥	27/53 (50.9, 36.8 – 64.9)	14	46.7 (28.3 – 65.7)	13	56.5 (34.5 – 76.8)	21	**67.7 (48.6 – 83.3)**	6	27.3 (10.7 – 50.2)
Not undertaken health screening (blood, bowel, cervix, or prostate)	0/53 (0.0, 0.00 – 6.7)	0	0.0 (0.0 – 11.6)	0	0.0 (0.0 – 14.8)	0	0.0 (0.0 – 11.2)	0	0.0 (0.0 – 15.4)

¥ $p<.05$ for age or gender; ¥¥ $p<.05$ for both age and gender; *based on Australian recommendations [60]; **a little or much worse; ***once or more in last 12 months; ǂ numbers reporting fairly/very often/always; 95% CI: 95% confidence interval (Clopper-Pearson exact); D: OHIP-14 health domain; FMS: functional movement scale; GPCog: General Practitioner Assessment of Cognition; K10: Kessler Psychological Distress Scale; L: litres; m/s: metres per second; OHIP-14: Oral Health Impact Profile-14; PFB: pelvic floor bother; PSQI: Pittsburgh Sleep Quality Index; SD; standard deviation of the proportion

Supplementary Table 3. Health assessments, by gender and age group differences (significantly more likely highlighted bold)

	Overall		Male		Female		Less than 55 years		55 years and over	
	m	95% CI, SD	m	95% CI, SD	m	95% CI, SD	m	95% CI, SD	m	95% CI, SD
Strength and dexterity objective measurement)										
Grip strength standing **¥	32.0	29.1 – 34.9, 9.3	**37.6**	**34.0 – 41.2, 8.1**	25.9	22.9 – 28.9, 6.3	33.7	30.4 – 37.1, 8.3	29.3	23.7 – 34.9, 10.5
Grip strength sitting **¥	30.7	27.9 – 33.5, 9.4	**36.0**	**32.4 – 39.6, 8.5**	24.6	21.8 – 27.4, 6.1	32.3	28.7 – 35.9, 9.1	28.3	23.5 – 33.0, 9.5
Purdue Pegboard dexterity										
Both hands (30 seconds)	7.9	6.8 – 9.0, 3.8	7.6	6.2 – 9.1, 3.7	8.2	6.5 – 10.0, 4.0	8.6	7.1 – 10.1, 3.9	6.8	5.2 – 8.5, 3.4
Sum (right + left = both)	28.4	25.9 – 30.9, 8.7	27.1	23.7 – 30.5, 8.7	30.0	26.2 – 33.9, 8.7	31.0	27.9 – 34.2, 8.4	24.4	20.8 – 28.1, 7.8
Assembly alternate hands (60 seconds) ¥	3.8	3.4 – 4.3, 1.4	3.7	3.2 – 4.1, 1.2	4.1	3.3 – 4.8, 1.7	**4.4**	**3.9 – 4.8, 1.3**	3.0	2.4 – 3.6, 1.3
Six-minute-walk (m/s) **¥	0.7	0.7 – 0.7, 0.1	0.7	0.6 – 0.8, 0.1	0.8	0.7 – 0.9, 0.1	**0.8**	**0.7 – 0.9, 0.1**	0.6	0.5 – 0.7, 0.1

¥ p<.05 for age or gender; 95% CI: 95% confidence interval (Clopper-Pearson exact); SD; standard deviation
